# Peripheral blood monocyte-to-lymphocyte ratio at study enrollment predicts efficacy of the RTS,S malaria vaccine: analysis of pooled phase II clinical trial data

**DOI:** 10.1186/1741-7015-11-184

**Published:** 2013-08-21

**Authors:** George M Warimwe, Helen A Fletcher, Ally Olotu, Selidji T Agnandji, Adrian VS Hill, Kevin Marsh, Philip Bejon

**Affiliations:** 1 The Jenner Institute, University of Oxford, Old Road Campus Research Building, Roosevelt Drive, Oxford OX3 7DQ, United Kingdom; 2 Kenya Medical Research Institute-Wellcome Trust Research Programme, P.O. Box 230–80108, Kilifi, Kenya; 3 Medical Research Unit, Albert Schweitzer Hospital, B.P. 118, Lambarene, Gabon; 4 Institute of Tropical Medicine, University of Tübingen, Tübingen, Germany; 5 Centre for Clinical Vaccinology and Tropical Medicine, University of Oxford, Oxford OX3 7LJ, United Kingdom

**Keywords:** Malaria, Vaccine, Monocyte-to-lymphocyte ratio

## Abstract

**Background:**

RTS,S is the most advanced candidate malaria vaccine but it is only partially protective and the causes of inter-individual variation in efficacy are poorly understood. Here, we investigated whether peripheral blood monocyte-to-lymphocyte ratios (ML ratio), previously shown to correlate with clinical malaria risk, could account for differences in RTS,S efficacy among phase II trial participants in Africa.

**Methods:**

Of 11 geographical sites where RTS,S has been evaluated, pre-vaccination ML ratios were only available for trial participants in Kilifi, Kenya (N = 421) and Lambarene, Gabon (N = 189). Using time to first clinical malaria episode as the primary endpoint we evaluated the effect of accounting for ML ratio on RTS,S vaccine efficacy against clinical malaria by Cox regression modeling.

**Results:**

The unadjusted efficacy of RTS,S in this combined dataset was 47% (95% confidence interval (CI) 26% to 62%, *P* <0.001). However, RTS,S efficacy decreased with increasing ML ratio, ranging from 67% (95% CI 64% to 70%) at an ML ratio of 0.1 to 5% (95% CI -3% to 13%) at an ML ratio of 0.6. The statistical interaction between RTS,S vaccination and ML ratio was still evident after adjustment for covariates associated with clinical malaria risk in this dataset.

**Conclusion:**

The results suggest that stratification of study participants by ML ratio, easily measured from full differential blood counts before vaccination, might help identify children who are highly protected and those that are refractory to protection with the RTS,S vaccine. Identifying causes of low vaccine efficacy among individuals with high ML ratio could inform strategies to improve overall RTS,S vaccine efficacy.

**Trial registration:**

ClinicalTrials.Gov numbers NCT00380393 and NCT00436007

## Background

*Plasmodium falciparum* malaria is a major cause of childhood morbidity and mortality in sub-Saharan Africa [1,2]. An effective vaccine to complement existing disease control strategies is urgently needed. RTS,S, currently in phase III trials in 6- to 12-week-old infants and 5- to 17-month-old children in Africa [3], is the most advanced *P. falciparum* malaria vaccine candidate but it is only partially protective. In previous phase II trials conducted across 11 geographical sites in Africa, RTS,S efficacy ranged between 34% and 65% [4-13]. Pooled analysis of these phase II studies, as well as preliminary phase III data, found that RTS,S efficacy varied between individuals according to age at vaccination [3,14] and the intensity of malaria transmission [15,16].

We have previously shown that a high ratio of monocytes to lymphocytes (ML ratio) in peripheral blood at cross-sectional survey correlates with increased susceptibility to clinical malaria in older children (median age 4.5 years) during follow-up [17]. This correlation between ML ratio and clinical malaria risk was evident even after accounting for inter-individual differences in the levels of antibody correlates of clinical immunity in the study population [17]. We here investigated whether ML ratio measured before vaccination could account for inter-individual variation in RTS,S vaccine efficacy using published phase II data.

## Methods

### Study setting and participants

This study was reported according to the Strengthening the Reporting of Observational Studies in Epidemiology (STROBE) guidelines (see Additional file 1). The main aim of this study was to relate pre-vaccination ML ratios to RTS,S vaccine efficacy from published phase II clinical trials in Africa. We, therefore, sought to use pre-vaccination ML ratios and efficacy data from all 11 geographical sites in Africa where RTS,S has been evaluated in phase II clinical trials [16]. Full blood counts, including absolute lymphocyte count, were available for all sites. However, most sites did not collect absolute monocyte counts. Instead, they used cell counters that returned lymphocyte count, neutrophil count and a mixed cell count composed of the sum of monocytes, basophils and eosinophils. Our analysis was, therefore, restricted to Kilifi, Kenya [11] and Lambarene, Gabon [12], where absolute peripheral blood lymphocyte and monocyte counts were collected as distinct cell populations. These clinical trials are registered at ClinicalTrials.gov, number NCT00380393 for Kilifi, Kenya and NCT00436007 for Lambarene, Gabon. At both sites the RTS,S vaccine was co-administered with the AS01E adjuvant. The respective local and national ethics committees at both trial sites granted ethical approval for the studies as detailed in the primary publications [11,12]. Written informed consent was obtained from parents or guardians of all study participants.

The study in Kilifi, Kenya was a phase II double blind, randomized control trial of RTS,S safety, immunogenicity and efficacy when administered in a zero-, one-, two-month schedule, with a licensed rabies vaccine used for the control group [11]. This was a multi-center study of 894 children aged 5 to 17 months at first vaccination, 447 of who were from Kilifi, Kenya and 447 from Korogwe, Tanzania [11]. Recruitment for screening was done after public meetings and invitations in the respective communities. Children with any clinical illness, abnormal blood tests (including full differential blood count) and severe malnutrition were ineligible for the study. Vaccinations were performed between March and August 2007. Clinical malaria episodes (defined as an axillary temperature of ≥37°C accompanied by >2,500 *P. falciparum* parasites per μl of blood) were monitored by active surveillance through weekly home visits by field-workers beginning 2.5 months after the first vaccination [11]. Only children from Kilifi, Kenya were included in the present analysis and the median of the maximum follow-up duration per child was 14 months (interquartile range (IQR) 11.8 to 14.8 months) [11].

In Gabon, a randomized, open label trial design was used to evaluate safety, immunogenicity and efficacy of RTS,S among infants aged 6 to 10 weeks at first vaccination. This was also a multi-center study involving a total of 511 infants of whom 220 were from Lambarene, Gabon, and the rest from Bagamoyo, Tanzania (N = 210) and Kintampo, Ghana (N = 81) [12]. Recruitment for screening was done following community-based information programs in Lambarene and Bagamoyo, while an ongoing demographic surveillance system that includes monitoring for births was used at Kintampo [18]. Children with any clinical or laboratory evidence (including full differential blood count) of any acute or chronic illness were ineligible, and all children must have had an oral polio vaccine and Bacillus Calmette–Guérin (BCG) vaccine as part of the immunization program for each country [18]. An aim of that study was to determine the feasibility of incorporating RTS,S into the Extended Program on Immunization (EPI) schedule in infants. Thus, infants either received the prescribed EPI vaccines alone (control group) or EPI vaccines in co-administration with RTS,S [12]. Vaccinations were performed in either a zero-, one-, two-month schedule or zero-, one-, seven-month schedule over an eight-month period from April 2007 during a safety and immunogenicity assessment of RTS,S in infants [18]. For assessment of efficacy, passive surveillance for clinical malaria episodes whereby parents/guardians had the responsibility to report to a health facility if their child was ill was done beginning two weeks after the final vaccination. Clinical malaria was defined as an axillary temperature of ≥37°C accompanied by a lower parasitemia threshold of >500 *P. falciparum* parasites per μl of blood [12]. This was to account for the younger age and lower levels of naturally acquired immunity in the studied age group (infants aged 6 to 10 weeks at first vaccination) as has been discussed in previous clinical malaria case definition studies [19]. Only children from Lambarene, Gabon were considered in the present analysis and the median of the maximum follow-up duration per child was 12 months (IQR 11.1 to 14.5 months) [12].

The median time interval between measurement of ML ratio at screening and vaccination was 57 days for Kenya and 60 days for Gabon, but this was not expected to confound observed associations since we have previously shown that ML ratios among healthy children are stable over time [17].

### Statistical analysis

ML ratio was defined as the ratio between the absolute peripheral blood monocyte count and lymphocyte count [17] both acquired using a Coulter counter on blood sampled at screening before receiving any vaccine. Vaccine efficacy was defined as 1 minus the hazard ratio (HR) following Cox regression modeling with time to the first or only episode of clinical malaria as the primary endpoint [11,12]. To estimate RTS,S vaccine efficacy at different levels of ML ratio we tested for a statistical interaction between pre-vaccination ML ratio and RTS,S vaccination by Cox regression modeling, with the trial site included as a fixed effect. We used the multivariable fractional polynomial method to estimate the linear and non-linear effects of ML ratio and its interaction with RTS,S vaccination, but found no evidence to support the use of a model accounting for non-linearity (*P* = 0.2). We used non-parametric methods to assess the relationship between pre-vaccination ML ratios and the following variables: 1) RTS,S-induced peak IgG antibody levels, measured by enzyme-linked immunosorbent assay (ELISA) three months after the first vaccination, 2) RTS,S-induced T cell responses, measured by flow cytometry 12 months after the final vaccination, 3) age at vaccination, 4) insecticide-treated bed net use, 5) distance from a health facility, and 6) a weighted local parasite exposure index [20]. With the exception of bed net use, where the Mann–Whitney *U* test was used, all covariates were continuous variables and Spearman’s rank correlation coefficient was used for their univariate analyses. Of these covariates, only RTS,S-induced antibody response data and age at vaccination were available for both trial sites. All other variables were available for Kilifi, Kenya only. To account for their effect on the relationship between RTS,S efficacy and ML ratio all variables were included in a final Cox regression model and multivariable fractional polynomials used to exclude covariates with *P*-values >0.05 by backward elimination. Stata™ version 11 (StataCorp LP, College Station, Texas, USA) was used for all our additional analysis reported here and *P*-values <0.05 were considered statistically significant.

## Results and discussion

A total of 667 children at both trial sites were randomly assigned to the RTS,S group or the control group in the original phase II studies. Of these, pre-vaccination ML ratios were only available for 610 children (338 in the RTS,S group and 272 in the control group) to whom the present analysis is restricted. The median age of this subgroup of children at the time of vaccination was 8 months (7.5 in the RTS,S group and 10 in the control group). The median of the maximum follow-up duration per child was 13.5 months (13.6 in the RTS,S group and 13.4 in the control group). A total of 60 and 83 first or only clinical malaria episodes were reported in the RTS,S and control groups, respectively. The unadjusted efficacy of RTS,S against this primary endpoint of time to first clinical malaria episode in the combined dataset was 47% (95% CI 26% to 62%, *P* <0.001).

**Figure 1:**
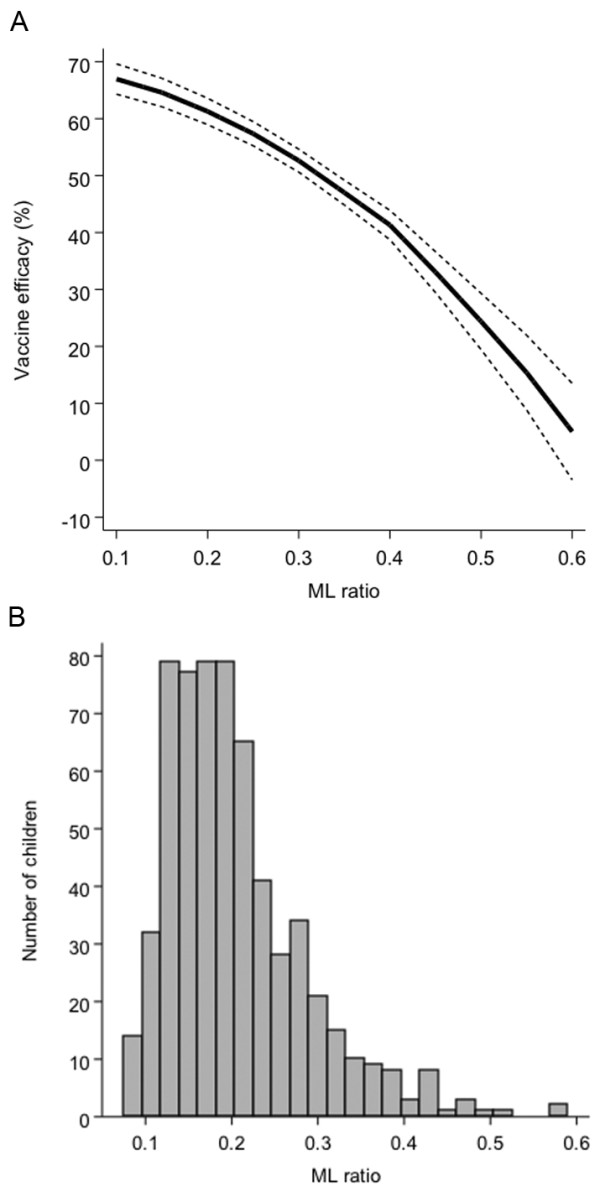
**RTS,S vaccine efficacy estimates at different levels of ML ratio measured before vaccination.** In **(A)**, the predicted efficacy (bold line) and 95% confidence intervals (dashed lines) of the RTS,S vaccine are shown in relation to the pre-vaccination ML ratio following bootstrap analysis of a Cox regression model predicting time to first clinical malaria episode with RTS,S vaccination, pre-vaccination ML ratio and an interaction term for both variables as covariates. In **(B)**, the distribution of pre-vaccination ML ratios for all the 610 children included in the study is shown.

**Figure 2:**
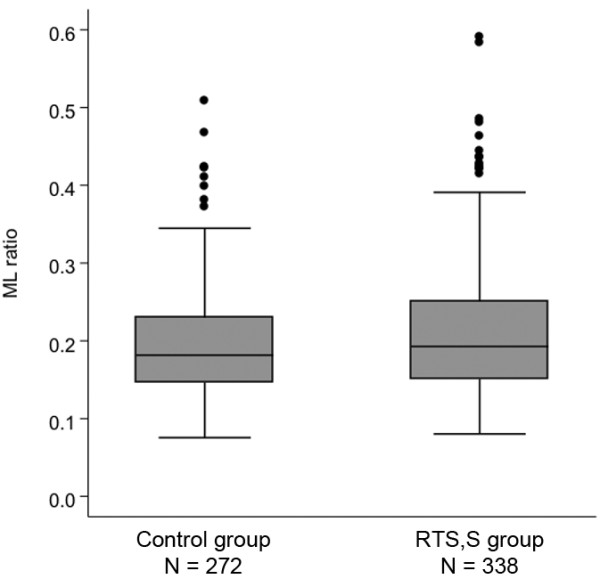
**Distribution of ML ratios among RTS,S vaccinees and controls.** Box-whisker plots are used to compare the distribution of ML ratios between study participants in the RTS,S and control groups, respectively. There was no significant difference in the ML ratio distribution between the two groups (Mann–Whitney *U* test z = -1.7, *P* = 0.1).

ML ratio did not directly correlate with clinical malaria risk among individuals in the RTS,S group (HR = 1.2, 95% CI 0.58 to 2.66, *P* = 0.6) or among controls (HR = 0.7, 95% CI 0.28 to 2.02, *P* = 0.6). However, there was strong evidence for a statistical interaction between ML ratio and vaccine efficacy (*P* = 0.006) suggesting that the protective effect of vaccination is significantly modified by ML ratio. RTS,S vaccine efficacy among children with an ML ratio of 0.1 was 67% (95% CI 64% to 70%) but only 5% (95% CI -3% to 13%) in those with an ML ratio of 0.6 (Figure 1). The distribution of ML ratios did not differ between the RTS,S and control groups thus ruling out any potential bias from such group differences in the vaccine efficacy estimates (Figure 2). A tendency towards an interaction between ML ratio and RTS,S vaccination was observed when the cohorts were analyzed separately but did not reach statistical significance (*P* = 0.08 for Kenya and *P* = 0.05 for Gabon).

As monocytes and lymphocytes play a crucial role in the induction and maintenance of an immune response, we determined if ML ratio correlated with antibody or T cell responses induced by the RTS,S vaccine. For this analysis, we used previously published antibody and T cell data [13,21] measured by ELISA and flow cytometry, respectively, using the constituent circumsporozoite (CS) protein of the RTS,S vaccine as an antigen. ML ratio showed no association with the peak RTS,S-induced IgG antibody response to the CS protein (rho = -0.06, *P* = 0.3; see Additional file 2), but the interaction between RTS,S and ML ratio was still evident after adjustment for this variable (*P* <0.001). No correlation was observed between ML ratio and the frequency of CD4+ or CD8+ T cells staining positive for IFNγ, IL-2 or TNFα on flow cytometry following restimulation of whole blood with overlapping peptide pools spanning the full length of CS protein (rho <0.1, *P* >0.05 for all). It remains possible that the ML ratio modifies RTS,S vaccine efficacy independent of the measured adaptive immune response or that it is an indirect marker of an as yet unidentified mechanism important for clinical protection. Though the effect of ML ratio on RTS,S efficacy does not appear to be through anti-CS protein IgG antibody quantity, we cannot rule out a role for antibody affinity and other functional properties of the induced response. It is noteworthy that the association between high ML ratio and clinical malaria risk in our previous longitudinal study of older children with naturally acquired immunity was independent of antibody correlates of clinical protection in the study population [17].

We next considered possible confounding by other covariates. ML ratio was significantly correlated with age at vaccination (available for both sites; rho = -0.14, *P* <0.001), but age was not associated with clinical malaria risk in our dataset (HR = 1.1, 95% CI 0.93 to 1.26, *P* = 0.3). We also examined correlations between ML ratio and covariates associated with clinical malaria risk in previous studies, namely use of an insecticide-treated bed net (Mann–Whitney *U* test z = 1.04, *P* = 0.3), distance from a health facility (rho = -0.10, *P* = 0.04) and a weighted local parasite exposure index [15,20] (rho = 0.05, *P* = 0.3), all available for Kenya only. The statistical interaction between RTS,S and pre-vaccination ML ratio was still evident (*P* <0.001) in a final model accounting for age, RTS,S immunogenicity, bed net use, proximity to health facility and parasite exposure index as covariates.

Together the results suggest that stratification of vaccine trial participants by ML ratio, easily measured from full differential blood counts at study enrollment, might help identify children who are highly protected and those that are refractory to protection with RTS,S. However, we do acknowledge several limitations in our analysis.

Pre-vaccination ML ratios were only available at 2 of 11 geographical sites where phase II clinical trials of RTS,S have been conducted. This clearly limits our ability to extend our interpretations to other RTS,S study populations. Further, while we assessed the effect of several potential confounders, not all covariates considered here were available at both trial sites. Nevertheless, it is encouraging that despite our relatively small sample size, the effect of ML ratio on RTS,S vaccine efficacy appears independent of age at vaccination and level of malaria parasite exposure, two key determinants of inter-individual variation in RTS,S vaccine efficacy identified in recent analysis of pooled phase II data and in preliminary analysis of phase III data from all 11 trial sites [3,14-16]. The much larger ongoing phase III trial of RTS,S in the same study populations should provide more conclusive evidence on the relationship between ML ratio and vaccine efficacy.

Whether interindividual variation in efficacy of other candidate malaria vaccines in development correlates with differences in pre-vaccination ML ratio remains an open question. However, recent studies on mouse models have demonstrated suppression of vaccine immunity by inflammatory monocytes and the enhancement of vaccine efficacy against tumors following monocyte depletion at the time of vaccination [22]. Further, inflammatory monocytes have been shown to accumulate and suppress anti-viral T cell responses during chronic lymphocytic choriomeningitis infection in mice [23]. It is plausible that RTS,S vaccine efficacy is specifically inhibited by inflammatory monocytes, thus confounding induction of an effective adaptive response, but further studies in both animal models and humans will be needed to confirm this.

## Conclusions

In summary, we find that variation in RTS,S vaccine efficacy between individuals can be attributed to differences in ML ratio measured before vaccination. Defining the underlying mechanism(s) for low vaccine efficacy among individuals with high ML ratio may help inform strategies to improve overall RTS,S vaccine efficacy, with expected benefits to the childhood population in Africa who bear the brunt of malarial morbidity and mortality.

## Abbreviations

BCG vaccine: Bacillus Calmette–Guérin vaccine; CS: Circumsporozoite; EPI: Extended Program on Immunization; IFNγ: Interferon gamma; IL: Interleukin; IQR: Interquartile range; ML ratio: Monocyte-to-lymphocyte ratio; TNFα: Tumor necrosis factor alpha.

## Competing interests

The authors declare that they have no competing interests.

## Authors’ contributions

GMW, HAF, AVSH and PB designed the study and were involved in data analysis and interpretation. AO, STA and KM were involved in data collection and interpretation. All authors contributed to writing and approved the final manuscript.

## Pre-publication history

The pre-publication history for this paper can be accessed here:

http://www.biomedcentral.com/1741-7015/11/184/prepub

## Supplementary Material

Additional file 1 STROBE checklist. Click here for file

Additional file 2 **Relationship between peak anti-circumsporozoite protein antibody titers and ML ratio.** Spearman’s rank correlation coefficient is used to assess the relationship between pre-vaccination ML ratios and the peak IgG antibody response to the circumsporozoite protein, presented as enzyme-linked immunosorbent assay unit (EU) per milliliter, among RTS,S vaccinees. Responses among children in the control group were very low or undetectable throughout follow-up. Click here for file
