# Supplementary material for: Peripheral blood monocyte-to-lymphocyte ratio at study enrollment predicts efficacy of the RTS,S malaria vaccine: analysis of pooled phase II clinical trial data
Source: BMC Med. 2013 Aug 21;11:184. doi: 10.1186/1741-7015-11-184 (PMC3765422; doi:10.1186/1741-7015-11-184)
Supplement: Additional file 1 — STROBE checklist. [file 1741-7015-11-184-S1.doc]

STROBE Statement—Checklist of items that should be included in reports of ***cohort studies***

|  | Item No | Recommendation |
| --- | --- | --- |
| **Title and abstract** | 1 | (*a*) **Peripheral blood monocyte-to-lymphocyte ratio at study enrolment predicts efficacy of the RTS,S malaria vaccine: analysis of pooled phase 2 clinical trial data** |
|  |  | (*b*) **Background.** RTS,S is the most advanced candidate malaria vaccine but it is only partially protective and the causes of inter-individual variation in efficacy are poorly understood. Here, we investigated whether peripheral blood monocyte-to-lymphocyte ratios (ML ratio), previously shown to correlate with clinical malaria risk, could account for differences in RTS,S efficacy among phase II trial participants in Africa.  **Methods.** Of eleven geographical sites where RTS,S has been evaluated, pre-vaccination ML ratios were only available for trial participants in Kilifi, Kenya (N=421) and Lambarene, Gabon (N=189). Using time to first clinical malaria episode as the primary endpoint we evaluated the effect of accounting for ML ratio on RTS,S vaccine efficacy against clinical malaria by Cox regression modeling.  **Results.** The unadjusted efficacy of RTS,S in this combined dataset was 47% (95% confidence interval (CI) 26% to 62%, p<0.001). However, RTS,S efficacy decreased with increasing ML ratio, ranging from 67% (95% CI 64% to 70%) at an ML ratio of 0.1 to 5% (95% CI -3% to 13%) at an ML ratio of 0.6. The statistical interaction between RTS,S vaccination and ML ratio was still evident after adjustment for covariates associated with clinical malaria risk in this dataset.  **Conclusion.** The results suggest that stratification of study participants by ML ratio, easily measured from full differential blood counts before vaccination, might help identify children who are highly protected and those that are refractory to protection with the RTS,S vaccine. Identifying causes of low vaccine efficacy among individuals with high ML ratio could inform strategies to improve overall RTS,S vaccine efficacy.  **Trial registration.** ClinicalTrials.Gov numbers NCT00380393 and NCT00436007. |
| Introduction | | |
| Background/rationale | 2 | *Plasmodium falciparum* malaria is a major cause of childhood morbidity and mortality in sub-Saharan Africa . An effective vaccine to complement existing disease control strategies is urgently needed. RTS,S, currently in phase III trials in 6-12 week old infants and 5-17 month old children in Africa , is the most advanced *P. falciparum* malaria vaccine candidate but it is only partially protective. In previous phase II trials conducted across eleven geographical sites in Africa RTS,S efficacy ranged between 34% and 65% . Pooled analysis of these phase II studies, as well as preliminary phase III data, found that RTS,S efficacy varied between individuals according to age at vaccination and the intensity of malaria transmission .  We have previously shown that a high ratio of monocytes to lymphocytes (ML ratio) in peripheral blood at cross-sectional survey correlates with increased susceptibility to clinical malaria in older children (median age 4.5 years) during follow-up . This correlation between ML ratio and clinical malaria risk was evident even after accounting for inter-individual differences in the levels of antibody correlates of clinical immunity in the study population . We here investigated whether ML ratio measured before vaccination could account for inter-individual variation in RTS,S vaccine efficacy using published phase II data. |
| Objectives | 3 | The main aim of this study was to evaluate the relationship pre-vaccination ML ratios to RTS,S vaccine efficacy using published data from phase II clinical trials in Africa. |
| Methods | | |
| Study design | 4 | We sought to use pre-vaccination ML ratios and efficacy data from all eleven geographical sites in Africa where RTS,S has been evaluated in phase II clinical trials. However, absolute peripheral blood monocyte and lymphocyte counts, both required for calculation of ML ratio, were only available for trial participants in Kilifi, Kenya and Lambarene, Gabon thus restricting our analysis to these sites. |
| Setting | 5 | These clinical trials are registered at ClinicalTrials.gov, number NCT00380393 for Kilifi, Kenya and NCT00436007 for Lambarene, Gabon. At both sites the RTS,S vaccine was co-administered with the AS01E adjuvant. The respective local and national ethics committees at both trial sites granted ethical approval for the studies as detailed in the primary publications . Written informed consent was obtained from parents or guardians of all study participants.  The study in Kilifi, Kenya was a phase II double blind randomized control trial of RTS,S safety, immunogenicity and efficacy when administered in a 0, 1, 2 month schedule, with a licensed rabies vaccine used for the control group . This was a multi-center study of 894 children aged 5 to 17 months old at first vaccination, 447 of who were from Kilifi, Kenya and 447 from Korogwe, Tanzania . Recruitment for screening was done after public meetings and invitations in the respective communities. Children with any clinical illness, abnormal blood tests (including full differential blood count) and severe malnutrition were ineligible for the study. Vaccinations were performed between March and August 2007. Clinical malaria episodes (defined as an axillary temperature of ≥37°C accompanied by >2500 *P. falciparum* parasites per μl of blood) were monitored by active surveillance through weekly home visits by field-workers beginning 2.5 months after the first vaccination . Only children from Kilifi, Kenya were included in the present analysis and the median of the maximum follow-up duration per child was 14 months (interquartile range (IQR) 11.8 to 14.8 months) .  In Gabon, a randomized open label trial design was used to evaluate safety, immunogenicity and efficacy of RTS,S among infants aged 6 to 10 weeks old at first vaccination. This was also a multi-center study involving a total of 511 infants of who 220 were from Lambarene, Gabon, and the rest from Bagamoyo, Tanzania (N=210) and Kintampo, Ghana (N=81) . Recruitment for screening was done following community-based information programs in Lambarene and Bagamoyo, while an ongoing demographic surveillance system that includes monitoring for births was used at Kintampo . Children with any clinical or laboratory evidence (including full differential blood count) of any acute or chronic illness were ineligible, and all children must have had an oral polio vaccine and BCG vaccine as part of the immunization programme for each country . An aim of that study was to determine the feasibility of incorporating RTS,S into the Extended Program on Immunization (EPI) schedule in infants. Thus, infants either received the prescribed EPI vaccines alone (control group) or EPI vaccines in co-administration with RTS,S . Vaccinations were performed in either a 0, 1, 2 month schedule or 0, 1, 7 month schedule over an eight-month period as from April 2007 during a safety and immunogenicity assessment of RTS,S in infants . For assessment of efficacy, passive surveillance for clinical malaria episodes whereby parents/guardians had the responsibility to report to a health facility if their child was ill was done beginning two weeks after the final vaccination. Clinical malaria was defined as an axillary temperature of ≥37°C accompanied by a lower parasitaemia threshold of >500 *P. falciparum* parasites per μl of blood . This was to account for the younger age and lower levels of naturally acquired immunity in the studied age group (infants aged 6-10 weeks at first vaccination) as has been discussed in previous clinical malaria case definition studies . Only children from Lambarene, Gabon were considered in the present analysis and the median of the maximum follow-up duration per child was 12 months (IQR 11.1 to 14.5 months) . |
| Participants | 6 | (*a*) **Kenya**: Children aged 5-17 months old and with no history or clinical or laboratory evidence of any significant illness or severe malnutrition were eligible for the study. Recruitment for screening was done after public meetings and invitations in the respective communities. Clinical malaria episodes were monitored by active surveillance through weekly home visits by field-workers beginning 2.5 months after the first vaccination . **Gabon**: Infants aged 6-10 weeks were eligible for the study. Exclusion criteria inlcuded any clinical or laboratory evidence of any acute or chronic illness. Further, all children must have had an oral polio vaccine and BCG vaccine as part of the immunization programme for each country . Recruitment for screening was done following community-based information programs in Lambarene and Bagamoyo, while an ongoing demographic surveillance system that includes monitoring for births was used at Kintampo . Passive surveillance for clinical malaria episodes was used. Thus, parents/guardians had the responsibility to report to a health facility if their child was ill beginning two weeks after the final vaccination. |
|  |  | (*b*) Eligible children were randomised to receive RTS,S or control vaccine in a 1:1 ratio at both Kilifi and Korogwe in the 5-17 month old children study. In Lambarene, Bagamoyo and Kintampo infants received EPI vaccines only, or EPI vaccines together with RTS,S in a 0, 1, 2 month schedule or a 0, 1, 7 schedule in a 1:1:1 ratio at all three study sites. |
| Variables | 7 | ML ratio was defined as the ratio between the absolute peripheral blood monocyte count and lymphocyte count , both acquired using a coulter counter on blood sampled at screening before receiving any vaccine. Vaccine efficacy was defined as 1 minus the hazard ratio (HR) following Cox regression modelling with time to the first or only episode of clinical malaria as the primary endpoint . Potential confounders considered were 1) RTS,S-induced peak IgG antibody levels, measured by enzyme-linked immunosorbent assay (ELISA) three months after the first vaccination, 2) RTS,S-induced T cell responses, measured by flow cytometry 12 months after the final vaccination, 3) age at vaccination, 4) insecticide-treated bed net use, 5) distance from a health facility and, 6) a weighted local parasite exposure index . |
| Data sources/ measurement | 8* | We used published data for all our analysis. ML ratios were derived from full differential blood counts acquired using a coulter counter. RTS,S-induced antibodies and T cell responses were measured by ELISA and flow cytometry, respectively. Proximity to health facility and the weighted local parasite exposure index were obtained from an ongoing demographic health surveillance system in Kilifi. |
| Bias | 9 | We confirmed that the distribution of ML ratios between RTS,S vaccinees and the control group were comparable, thus ruling out such differences as a source of bias in our analysis. |
| Study size | 10 | This retrospective analysis utilised published data. Thus, our sample size was dependent on availability of ML ratio data across the study sites. Only 2 of 11 sites had data available. |
| Quantitative variables | 11 | With the exception of bed net use, which was used as a binary variable, all covariates were continuous variables. |
| Statistical methods | 12 | (*a*) Cox regression modelling was used to assess vaccine efficacy, defined as 1 minus the hazard ratio, against the first or only clinical malaria episode. Mann-Whitney U test was used for univariate comparisons between binary and continuous variables. Where both variables were continuous the Spearman’s rank correlation coefficient was used for univariate comparisons. Multivariable fractional polynomials were used to allow for linear and non-linear effects and for backward elimination of variables in the Cox regression modelling. |
|  |  | (*b*) To estimate RTS,S vaccine efficacy at different levels of ML ratio we tested for a statistical interaction between pre-vaccination ML ratio and RTS,S vaccination by Cox regression modelling, with trial site included as a fixed effect. |
|  |  | (*c*) Only children with ML ratio data were included in the analysis. |
|  |  | (*d*) Survival analysis was used hence accounting for each individual’s duration in the study |
|  |  | (*e*) Bootstrap resampling was used to estimate the effect of ML ratio on vaccine efficacy. |
| Results | | |
| Participants | 13* | (a) A total of 667 children at both trial sites were randomly assigned to the RTS,S group or control group in the original phase II studies. Of these, pre-vaccination ML ratios were only available for 610 children (338 in the RTS,S group and 272 in the control group) to whom the present analysis is restricted. |
|  |  | (b) Only children with ML ratio data were included in this analysis: RTS,S group = 338, Control group = 272 |
|  |  | (c) N/A |
| Descriptive data | 14* | (a) 338 received RTS,S and 272 were in the control group. The median age of this subgroup of children at the time of vaccination was: RTS,S group 7.5 months (IQR 1.6 to 12 months), Control group 10 months (IQR 5 to 12 months). The distribution of ML ratios did not differ between the RTS,S (median 0.19, IQR 0.15 to 0.25) and control (median 0.18, IQR 0.15 to 0.23) groups. |
|  |  | (b) Only children with ML ratio who were randomised to the RTS,S or control group and who contributed to follow-up (total number = 610 of who 338 were in RTS,S group) were included in the main analysis of this retrospective study of published data. For the subgroup and covariate analyses all children from Gabon missed data on bed-net use, parasite exposure, proximity to health facility and RTS,S-induced T cell responses as none of these variables were recorded/acquired at that site. |
|  |  | (c) The median of the maximum follow-up duration per child was: RTS,S group 13.6 months (IQR 11.6 to 14.9 months), Control group 13.4 months (IQR 10.7 to 14.7 months). |
| Outcome data | 15* | Number of subjects: RTS,S group= 338, number of events = 60; Control group= 272, number of events = 83 |
| Main results | 16 | (*a*) RTS,S had an unadjusted efficacy of 47% (95% CI 26% to 62%, p<0.001) against the primary endpoint of time to first clinical malaria episode in this combined dataset. ML ratio did not directly influence clinical malaria risk in the RTS,S group (HR=1.2, 95% CI 0.58 to 2.66, p=0.6) or among controls (HR=0.7, 95% CI 0.28 to 2.02, p=0.6), but there was a strongly significant interaction between ML ratio and vaccine efficacy (p=0.006). RTS,S vaccine efficacy among children with an ML ratio of 0.1 was 67% (95% CI 64% to 70%) but only 5% (95% CI -3% to 13%) in those with an ML ratio of 0.6. |
|  |  | (*b*) N/A |
|  |  | (*c*) N/A |
| Other analyses | 17 | ML ratio showed a positive correlation with the peak RTS,S-induced IgG antibody response to the CS protein (rho=0.15, p=0.009), but the interaction between RTS,S and ML ratio was still evident after adjustment for this variable (p<0.001). No correlation was observed between ML ratio and the frequency of CD4+ or CD8+ T cells staining positive for IFNγ, IL-2 or TNFα on flow cytometry following restimulation of whole blood with overlapping peptide pools spanning the full length of CS protein (rho<0.1, p>0.05 for all). ML ratio was significantly correlated with age at vaccination (available for both sites; rho=-0.14, p<0.001), but age was not associated with clinical malaria risk in our dataset (HR=1.1, 95% CI 0.93 to 1.26, p=0.3). We also examined correlations between ML ratio and covariates associated with clinical malaria risk in previous studies, namely use of an insecticide treated bed-net (Mann-Whitney U test z=1.04, p=0.3), distance from a health facility (rho=-0.10, p=0.04) and a weighted local parasite exposure index (rho=0.05, p=0.3), all available for Kenya only. The statistical interaction between RTS,S and pre-vaccination ML ratio was still evident (p<0.001) in a final model accounting for age, RTS,S immunogenicity, bed-net use, proximity to health facility and parasite exposure index as covariates. |
| Discussion | | |
| Key results | 18 | Together the results suggest that stratification of vaccine trial participants by ML ratio, easily measured from full differential blood counts at study enrolment, might help identify children who are highly protected and those that are refractory to protection with RTS,S. |
| Limitations | 19 | Our study had several limitations. Pre-vaccination ML ratios were only available at two of eleven geographical sites where phase II clinical trials of RTS,S have been conducted. This clearly limits our ability to extend our interpretations to other RTS,S study populations. Further, while we assessed the effect of potential confounders not all covariates considered here were available at both trial sites. Nevertheless, it is encouraging that despite our relatively small sample size, the effect of ML ratio on RTS,S vaccine efficacy appears independent of age at vaccination and level of malaria parasite exposure, two key determinants of inter-individual variation in RTS,S vaccine efficacy identified in recent analysis of phase II and preliminary phase III data from all eleven trial sites . The much larger ongoing phase III trial of RTS,S in the same study populations should provide more conclusive evidence on the relationship between ML ratio and vaccine efficacy. Whether inter-individual variation in efficacy of other candidate malaria vaccines in development correlates with differences in pre-vaccination ML ratio remains an open question. |
| Interpretation | 20 | In summary we find that variation in RTS,S vaccine efficacy between individuals can be attributed to differences in ML ratio measured before vaccination. Defining the underlying mechanism(s) for low vaccine efficacy among individuals with high ML ratio may help inform strategies to improve overall RTS,S vaccine efficacy, with expected benefits to the childhood population in Africa who bear the brunt of malarial morbidity and mortality. |
| Generalisability | 21 | That pre-vaccination ML ratios were only available at two of eleven geographical sites where phase II clinical trials of RTS,S have been conducted limits our ability to extend our interpretations to other RTS,S study populations. The ongoing phase III clinical trial will help address this, where ML ratio data has been collected. In addition, whether ML ratio can predict efficacy of other candidate malaria vaccines in development will need to be evaluated in future studies. |
| Other information | | |
| Funding | 22 | GMW is supported by a Wellcome Trust fellowship (grant number 098635/B/12/Z). Conduct of the reported trials and generation of the analysed trial data were funded by Program for Appropriate Technology in Health (PATH) Malaria Vaccine Initiative and GlaxoSmithKline (GSK) Biologicals. GSK Biologicals employees were co-investigators in the original phase II studies and their role in these studies is detailed in the primary publications. However, the funders had no role in the analysis presented here and the corresponding author had final responsibility of the decision to submit the manuscript for publication. |

*Give information separately for exposed and unexposed groups.

**Note:** An Explanation and Elaboration article discusses each checklist item and gives methodological background and published examples of transparent reporting. The STROBE checklist is best used in conjunction with this article (freely available on the Web sites of PLoS Medicine at http://www.plosmedicine.org/, Annals of Internal Medicine at http://www.annals.org/, and Epidemiology at http://www.epidem.com/). Information on the STROBE Initiative is available at http://www.strobe-statement.org.

**REFERENCES**:

1. Snow RW, Amratia P, Kabaria CW, Noor AM, Marsh K: **The changing limits and incidence of malaria in Africa: 1939-2009**. *Advances in parasitology* 2012, **78**:169-262.

2. World Health Organization: **World malaria report**. 2011.

3. Agnandji ST, Lell B, Soulanoudjingar SS, Fernandes JF, Abossolo BP, Conzelmann C, Methogo BG, Doucka Y, Flamen A, Mordmuller B *et al*: **First results of phase 3 trial of RTS,S/AS01 malaria vaccine in African children**. *The New England journal of medicine* 2011, **365**(20):1863-1875.

4. Abdulla S, Oberholzer R, Juma O, Kubhoja S, Machera F, Membi C, Omari S, Urassa A, Mshinda H, Jumanne A *et al*: **Safety and immunogenicity of RTS,S/AS02D malaria vaccine in infants**. *The New England journal of medicine* 2008, **359**(24):2533-2544.

5. Alonso PL, Sacarlal J, Aponte JJ, Leach A, Macete E, Milman J, Mandomando I, Spiessens B, Guinovart C, Espasa M *et al*: **Efficacy of the RTS,S/AS02A vaccine against Plasmodium falciparum infection and disease in young African children: randomised controlled trial**. *Lancet* 2004, **364**(9443):1411-1420.

6. Aponte JJ, Aide P, Renom M, Mandomando I, Bassat Q, Sacarlal J, Manaca MN, Lafuente S, Barbosa A, Leach A *et al*: **Safety of the RTS,S/AS02D candidate malaria vaccine in infants living in a highly endemic area of Mozambique: a double blind randomised controlled phase I/IIb trial**. *Lancet* 2007, **370**(9598):1543-1551.

7. Bojang KA, Milligan PJ, Pinder M, Vigneron L, Alloueche A, Kester KE, Ballou WR, Conway DJ, Reece WH, Gothard P *et al*: **Efficacy of RTS,S/AS02 malaria vaccine against Plasmodium falciparum infection in semi-immune adult men in The Gambia: a randomised trial**. *Lancet* 2001, **358**(9297):1927-1934.

8. Polhemus ME, Remich SA, Ogutu BR, Waitumbi JN, Otieno L, Apollo S, Cummings JF, Kester KE, Ockenhouse CF, Stewart A *et al*: **Evaluation of RTS,S/AS02A and RTS,S/AS01B in adults in a high malaria transmission area**. *PloS one* 2009, **4**(7):e6465.

9. Sacarlal J, Aide P, Aponte JJ, Renom M, Leach A, Mandomando I, Lievens M, Bassat Q, Lafuente S, Macete E *et al*: **Long-term safety and efficacy of the RTS,S/AS02A malaria vaccine in Mozambican children**. *The Journal of infectious diseases* 2009, **200**(3):329-336.

10. Alonso PL, Sacarlal J, Aponte JJ, Leach A, Macete E, Aide P, Sigauque B, Milman J, Mandomando I, Bassat Q *et al*: **Duration of protection with RTS,S/AS02A malaria vaccine in prevention of Plasmodium falciparum disease in Mozambican children: single-blind extended follow-up of a randomised controlled trial**. *Lancet* 2005, **366**(9502):2012-2018.

11. Bejon P, Lusingu J, Olotu A, Leach A, Lievens M, Vekemans J, Mshamu S, Lang T, Gould J, Dubois MC *et al*: **Efficacy of RTS,S/AS01E vaccine against malaria in children 5 to 17 months of age**. *The New England journal of medicine* 2008, **359**(24):2521-2532.

12. Asante KP, Abdulla S, Agnandji S, Lyimo J, Vekemans J, Soulanoudjingar S, Owusu R, Shomari M, Leach A, Jongert E *et al*: **Safety and efficacy of the RTS,S/AS01E candidate malaria vaccine given with expanded-programme-on-immunisation vaccines: 19 month follow-up of a randomised, open-label, phase 2 trial**. *The Lancet infectious diseases* 2011, **11**(10):741-749.

13. Olotu A, Lusingu J, Leach A, Lievens M, Vekemans J, Msham S, Lang T, Gould J, Dubois MC, Jongert E *et al*: **Efficacy of RTS,S/AS01E malaria vaccine and exploratory analysis on anti-circumsporozoite antibody titres and protection in children aged 5-17 months in Kenya and Tanzania: a randomised controlled trial**. *The Lancet infectious diseases* 2011, **11**(2):102-109.

14. Agnandji ST, Lell B, Fernandes JF, Abossolo BP, Methogo BG, Kabwende AL, Adegnika AA, Mordmuller B, Issifou S, Kremsner PG *et al*: **A phase 3 trial of RTS,S/AS01 malaria vaccine in African infants**. *The New England journal of medicine* 2012, **367**(24):2284-2295.

15. Olotu A, Fegan G, Wambua J, Nyangweso G, Awuondo KO, Leach A, Lievens M, Leboulleux D, Njuguna P, Peshu N *et al*: **Four-year efficacy of RTS,S/AS01E and its interaction with malaria exposure**. *The New England journal of medicine* 2013, **368**(12):1111-1120.

16. Bejon P, White MT, Olotu A, Bojang K, Lusingu JP, Salim N, Otsyula NN, Agnandji ST, Asante KP, Owusu-Agyei S *et al*: **Efficacy of RTS,S malaria vaccines: individual-participant pooled analysis of phase 2 data**. *The Lancet infectious diseases* 2013.

17. Warimwe GM, Murungi LM, Kamuyu G, Nyangweso GM, Wambua J, Naranbhai V, Fletcher HA, Hill AV, Bejon P, Osier FH *et al*: **The ratio of monocytes to lymphocytes in peripheral blood correlates with increased susceptibility to clinical malaria in kenyan children**. *PloS one* 2013, **8**(2):e57320.

18. Agnandji ST, Asante KP, Lyimo J, Vekemans J, Soulanoudjingar SS, Owusu R, Shomari M, Leach A, Fernandes J, Dosoo D *et al*: **Evaluation of the safety and immunogenicity of the RTS,S/AS01E malaria candidate vaccine when integrated in the expanded program of immunization**. *The Journal of infectious diseases* 2010, **202**(7):1076-1087.

19. Mwangi TW, Ross A, Snow RW, Marsh K: **Case definitions of clinical malaria under different transmission conditions in Kilifi District, Kenya**. *The Journal of infectious diseases* 2005, **191**(11):1932-1939.

20. Olotu A, Fegan G, Wambua J, Nyangweso G, Ogada E, Drakeley C, Marsh K, Bejon P: **Estimating individual exposure to malaria using local prevalence of malaria infection in the field**. *PloS one* 2012, **7**(3):e32929.
