# Supplementary figures and images for: Peripheral blood monocyte-to-lymphocyte ratio at study enrollment predicts efficacy of the RTS,S malaria vaccine: analysis of pooled phase II clinical trial data
Source: BMC Med. 2013 Aug 21;11:184. doi: 10.1186/1741-7015-11-184 (PMC3765422; doi:10.1186/1741-7015-11-184)

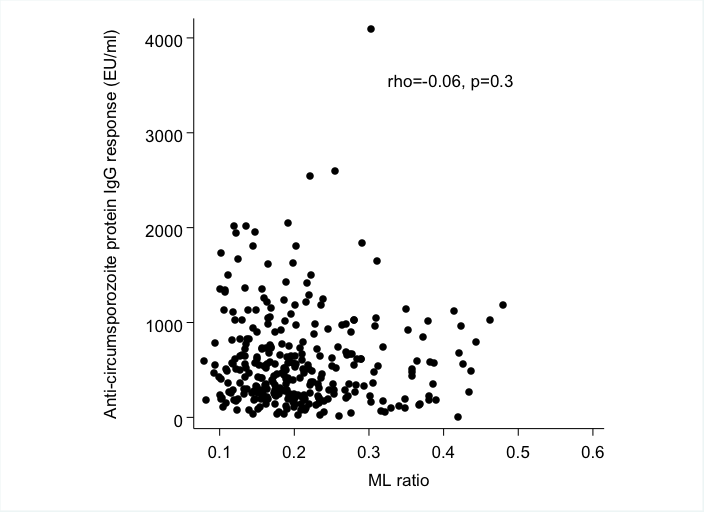

Supplement: Additional file 2 — Relationship between peak anti-circumsporozoite protein antibody titers and ML ratio. Spearman’s rank correlation coefficient is used to assess the relationship between pre-vaccination ML ratios and the peak IgG antibody response to the circumsporozoite protein, presented as enzyme-linked immunosorbent assay unit (EU) per milliliter, among RTS,S vaccinees. Responses among children in the control group were very low or undetectable throughout follow-up. [file 1741-7015-11-184-S2.tiff]
